# Efficacy of an educational manual for childbirth companions: pilot study of a randomized clinical trial

**DOI:** 10.1590/1518-8345.2277.2996

**Published:** 2018-05-07

**Authors:** Liana Mara Rocha Teles, Camila Félix Américo, Mônica Oliveira Batista Oriá, Camila Teixeira Moreira Vasconcelos, Odaléa Maria Brüggemann, Ana Kelve de Castro Damasceno

**Affiliations:** 1 PhD, Nursing. Adjunct Professor. Nursing Department, Universidade Federal do Ceará, Fortaleza, CE, Brazil.; 2 PhD, Nursing. Adjunct Professor. Nursing Department, Universidade Federal do Ceará, Fortaleza, CE, Brazil.; 3 PhD, Nursing. Associated Professor. Nursing Department, Universidade Federal do Ceará, Fortaleza, CE, Brazil. Research Productivity Scolarship CNPq.; 4 PhD, Nursing. Adjunct Professor. Nursing Department, Universidade Federal do Ceará, Fortaleza, CE, Brazil.; 5 PhD, in Tocogynecology. Associated Professor. Nursing Department, Universidade Federal do Ceará, Fortaleza, CE, Brazil. Research Productivity Scolarship CNPq.; 6 PhD, Nursing. Associated Professor. Nursing Department, Universidade Federal do Ceará, Fortaleza, CE, Brazil. Research Productivity Scolarship CNPq.

**Keywords:** Social Support, Parturition, Clinical Trial, Nursing, Health Education, Health Promotion

## Abstract

**Objective:**

to evaluate the effectiveness of an educational manual in the instrumentalization of companions to provide support to the parturients and check its influence on the satisfaction of companions and women during vaginal delivery.

**Method:**

pilot study of a randomized controlled clinical trial with 65 companions and puerperal women (intervention = 21 and control = 44). The previous knowledge of the companions was evaluated at baseline. The Evaluation Form for Companions in the Delivery Room was used to measure the actions provided and the satisfaction with the experience, and the Questionnaire for Evaluation of the Experience and Satisfaction of Puerperal Women with Labor and Delivery was used to evaluate the satisfaction of women with childbirth. The Student’s t-test or Wilcoxon, chi-square or Fisher’s exact test, risk ratios and 95% confidence intervals were used.

**Results:**

the companions in the intervention group performed a greater number of support actions (7.2 vs 4.6, p: 0.001) and had higher satisfaction scores (72.4 vs 64.2; p = 0.00). Puerperal women in the intervention group had higher satisfaction with childbirth (119.6 vs 107.9; p: 0.000).

**Conclusion:**

the manual was effective for the instrumentalization of companions, contributed to support actions to the parturients and had repercussions on the satisfaction of companions and women with the birthing process. RBR-776d9s

## Introduction

Childbirth is one of the most remarkable experiences in a woman’s life. It involves a mixture of sensations, feelings, desires, overcomings, and challenges that make it a complex, multidimensional process involving physiological and cognitive aspects. In this sense it is important that companions be prepared and well trained to participate in this moment, supporting and comforting the parturients and bringing greater satisfaction to the process of delivery and birth. Stimulating the participation of companions in delivery and birth is part of the qualification of humanized childbirth care^1^. 

Evidence shows that the continuous support from a companion who does not belong to the hospital’s professional team during the delivery provides several benefits for the woman and the newborn^2^ ^-^ ^4^. It is necessary, therefore, to develop and evaluate educational technologies for those who intend to participate in childbirth as companions, with the purpose of disseminating and expanding the knowledge about the physiology and care involved in the process of childbirth and techniques to support parturients. The lack of preparation of companions has been highlighted as one of the reasons for health institutions to prevent their presence^5^.

Based on the assumption that the development of educational technologies can contribute to the empowerment and better performance of companions in the delivery room, the manual entitled *“Preparing to be a companion during vaginal birth: what is important to know?”* ^6^. This educational technology seeks to encourage the development of skills in those who intend to participate in childbirth as companions. It is also an important tool to dynamize the methodology used by nurses in the systematization of their educational actions in the prenatal context. 

It is presumed that companions with access to the educational manual will be better prepared to provide support to parturients, bringing a positive effect on the satisfaction of companions and puerperal women with the birthing process. From that point on, the following question arose: will companions who have access to the educational manual during prenatal care provide more support to the parturients, leading to a greater satisfaction of companions and puerperal women with the process of childbirth? Thus, the objective of the present study was to evaluate the effectiveness of an educational manual in the instrumentalization of companions to provide support to the parturient women and to check its influence on the satisfaction of companions and women with the process of vaginal delivery.

## Method

This is a parallel, open, two-arm pilot Randomized Clinical Trial (RCT). Pilot studies are conducted to guide decisions on how to outline recruitment, gauging and intervention approaches and are particularly useful in studies on new forms of intervention^7^. In this sense, with the aim to evaluate a new educational technology and in view of the paucity of experimental studies assessing the impact of educational interventions on the performance of companions in the delivery room, a Pilot Study became necessary before the realization of a larger RCT. The methodology used was the *Consolidated Standards of Reporting Trials* (CONSORT) for Non-Pharmacological Interventions^8^.

The study was developed in two primary care insititutions in Fortaleza (CE). These Health Units were chosen because they are a reference to vaginal delivery of habitual risk and accept the presence of companions during the process of delivery.

The subjects of the study were the companions of women who underwent prenatal consultation in the Centro de Parto Normal Ligia Barros Costa (CPN-LBC) and the Centro Integrado de Educação e Saúde Casimiro José de Lima Filho (CIESCJLF) and the puerperal women who had the presence of a companion who participated in the prenatal care. The inclusion criteria for companions were: having been chosen by the pregnant woman to participate in delivery as a companion; having completed at least the fourth year of elementary school (level of schooling compatible with the readability index of the manual); and being companions of pregnant women with indication of vaginal delivery (type of delivery for which the manual is directed). The criterion of exclusion for companions was: prior experience as a companion during childbirth. The criterion of inclusion for puerperal women was: having had vaginal delivery; having had as a companion in the delivery room the same person approached in the first phase of this study. The criteria for discontinuing the participation of companions and puerperal women were: companions of pregnant women who progressed to cesarean section (elective/emergency); withdrawal from the study after the start of the collection; withdrawal or impossibility to be present at labor/delivery; choice of another companion at the moment of childbirth; change of address and/or telephone number that made the contact unfeasible after birth. Thus, the pairs (companion and puerperal woman) were selected and analyzed.

Since this is a pioneering Pilot Clinical Trial to evaluate the impact of an educational technology on the support provided by companions in the delivery room, the sample size was not calculated. Thus, the sample corresponded to all the companions (and the respective puerperae) recruited in the period, who met the inclusion criteria and who completed the follow-up, that is, went through all phases of the study. At the end, 65 companions, 21 in the Intervention Group (IG) and 44 in the Control Group (CG) were selected.

The participants were recruited by the field team and randomized into the IG or CG using sequence of random numbers generated at www.randomizer.org. The study was blinded to the field team responsible for evaluation phases III and IV, specified below. The IG was represented by the group of companions to whom the educational manual was made available. The CG corresponded to the group of companions eligible to participate in the survey who received the routine guidelines, characterized by individual guidelines during prenatal care and the course for pregnant women (and companions) promoted by the institutions. 

### *Data collection tools*

Three instruments were used for data collection (two for companions and one for the women). The *instrument 1* addressed the characterization of the companions and items for assessment of their prior knowledge about support techniques during childbirth. This instrument was applied to all eligible companions who agreed to participate in the survey. It is a *baseline* diagnostic tool (Phase 1). 

The *instrument 2*, the Evaluation Form for Companions in the Delivery Room, consisted of 22 questions and was applied to the companions during the Phase 3 to evaluate the support provided and the satisfaction with the experience in the delivery room. The instrument was composed of the following topics: support actions carried out; satisfaction in being a companion (labor and delivery); satisfaction with the support provided (labor and delivery); satisfaction with the way the birth process occurred; satisfaction with the delay (labor and delivery); satisfaction with the care provided by health professionals (labor and delivery); evaluation of the usefulness of the support provided and of the cooperation with health professionals. The score was distributed as follows: one point for each support action performed by the companion (questions 1 to 3) and a Likert-type response varying from one (none) to four (very much) points for the questions 4 to 22. These questions assessed the level of satisfaction of the companion with his or her experience. The final score of the instrument consisted of the sum of the number of support actions performed and the sum of the scores in the Likert-type questions. This instrument was prepared based on a previous study^6^ and evaluated by three researchers in the field of obstetrics.

 The *instrument 3* was a questionnaire entitled Evaluation of the Experience and Satisfaction of Puerperal Women with Labor and Delivery^9^. This questionnaire is divided into two parts: I. characterization of the puerperal women (items 1 to 13); and II. The short version of the Questionnaire of Experience and Satisfaction with Childbirth (QESC) (items 14 to 51). This instrument was applied to women during the Phase 4. 

The QESC has already been used and validated in a Brazilian study^10^ and is divided into 8 subscales, of which the following were selected for the present study: - Subscale 2 - Positive Experience, consisting of 22 items related to the confirmation of expectations, self-control, self-confidence, knowledge, pleasure and satisfaction with the experience of childbirth; - Subscale 3 - Negative experience, consisting of 12 items that refer to fear, malaise and pain during labor and delivery; - Subscale 4 - Relaxation, consisting of 6 items related to the experience of relaxation during labor and delivery; - Subscale 6 - Companion’s support, consisting of 8 items specifically related to the support of the companion. Items with negative topics such as pain, fear, malaise and worry have a reverse score.

 The QESC has a good internal consistency (Cronbach’s alpha = 0.9087) and test-retest fidelity index of 0.586^9^, allowing the consistent and reliable evaluation of the different dimensions that are relevant to the experience of childbirth.

### *Data colletion*

Data collection was performed in four phases, with three different teams of collaborators: one team responsible for Phase I, another responsible for Phase II and one responsible for Phases III and IV. The collaborators were previously trained and followed the guidelines of the Standard Operational Protocol developed for each phase of the study. The operationalization of the data collection took place as follows: 

*Phase I (Baseline):* interviews with companions to identify their sociodemographic profile and prior knowledge about support techniques. The form (Instrument 1) and Informed Consent Term (TCLE) were placed in sealed and numbered envelopes, which were randomized to either IG or CG;

*Phase II (Intervention):* after the randomization of the participants, the names and respective contacts of companions were given by the researcher to the team responsible for the intervention group. The companions selected for this group were invited to attend the institution, at a date and time previously scheduled. During the intervention, the educational manual was introduced and read, openig the possibility of interruption for expressing doubts or for making comments. A printed version of the manual was delivered to the participants and they were requested to keep the manual confidential, not lending or replicating this material in order to prevent the companions of the control group from having access to it, since this material is not yet a publication of public domain. Each intervention had an average duration of 20 minutes. 

The manual in question consists of 38 illustrations and 11 topics that deal sequentially from the preparation to go to the Maternity until the puerperal period. The topics covered in the handbook are: Few days before delivery (changes in the woman’s body that indicate the approach of delivery); Knowing the woman’s body (anatomy of the reproductive organs); Signs and symptoms of labor (events that indicate the onset of labor); Arriving at the maternity hospital (documents that should be brougth and professionals who can act in the delivery room, presenting the duties of each one of them); Techniques of pain relief at childbirth (benefit of each of the methods and how the companion can offer them to the woman); How does normal delivery happen? (the physiological mechanism of vaginal delivery); Rights and duties of the woman and the companion; and Notions of Citizenship (birth certificate and maternity and parental leave). The manual has already been evaluated by representatives of the target public and validated as to its appearance and content by specialists in the area of ​​women’s health and/or obstetrics^6^.

*Phase III (Evaluation of the support provided by the companion in the delivery room):* a telephone contact was made to the companion had already participated in labor and delivery (if the pregnant woman had not yet given birth, another call was made after one week). If the pregnant woman had progressed to a cesarean section or if her companion had not participated in the birth process, the reason that impeded the participation of the companion was recorded. If the participant had been at the delivery, the team applied the Instrument 2 (described above). 

*Phase IV (Satisfaction of the woman with the childbirth experience):* the Instrument 3 was used in this phase, also performed through a telephone contact. To evaluate the satisfaction of the woman with the process of childbirth, the following variables were considered: 1. to which extent the form of the labor process and the felt pain met her expectations; 2. to which extent was the woman able to relax and how useful was the relaxation provided; 3. how confident she felt and the situation under control; 4. to which extent she counted on the help of the companion and how useful that help was; 5. how much knowledge she had about the relative events during the birthing process; 6. level of fear, malaise, pleasure/satisfaction during the labor process; 7. how much she cooperated with health professionals; and 8. how much she remembers how painful the process of childbirth was; and the satisfaction with the form, time and intensity of pain during labor and delivery, variables corresponding to QESC Subsections 2, 3, 4 and 6. 

### *Evaluated outcomes*

The primary outcome was the support provided by the companion who used the educational manual, measured by the number of support actions (emotional, physical, informational and intermediation) provided by the companion to the parturient. The secondary outcomes were the satisfaction of the companion and the woman with the process of childbirth, as measured through the Instruments II and III. These indicators were used to evaluate the effectiveness of the educational manual.

The control variables were: sociodemographic data of the companion: sex, age, marital status, schooling and family income; participation of the companion in educational strategies during prenatal care; degree of kinship of the companion; sociodemographic variables of the puerperal woman: age, marital status, schooling, family income; obstetric variables: number of gestations, births, abortions, stillbirths, living children; numbers of prenatal consultations performed by the puerperal woman; and participation of the puerperal woman in educational activities carried out during the prenatal period. Before analyzing the outcomes of the study, the similarity between the groups and the existence of confounding factors were verified.

The data were analyzed using the *Statistical Package for the Social Sciences* (SPSS), version 20.0. The *Kolmogorov-Smirnov* (KS) was used to verify the normality of continuous data. The groups were compared at the baseline and after the intervention, in separate analyses. The chi-square and *Fisher exact* tests (categorical variables) and the *Student t-test* or *Mann-Whitney* test (continuous variables) were used in these comparisons. The correlations were evaluated by means of the *Spearman correlation* index. The Relative Risk (RR) and the 95% confidence intervals were calculated for the main dependent variables, with a critical alpha of 0.05 to determine the level of significance.

The study was approved by the Research Ethics Committee of the Federal University of Ceará (nº 576.174/14) and registered in the database of the Brazilian Clinical Trials Registry (ReBEC) (RBR-776d9s). The study participants signed an ICF, and their anonymity was assured, according to the norms of Resolution nº 466/12 of the National Health Council of the Ministry of Health.

## Results

**Figure 1:**
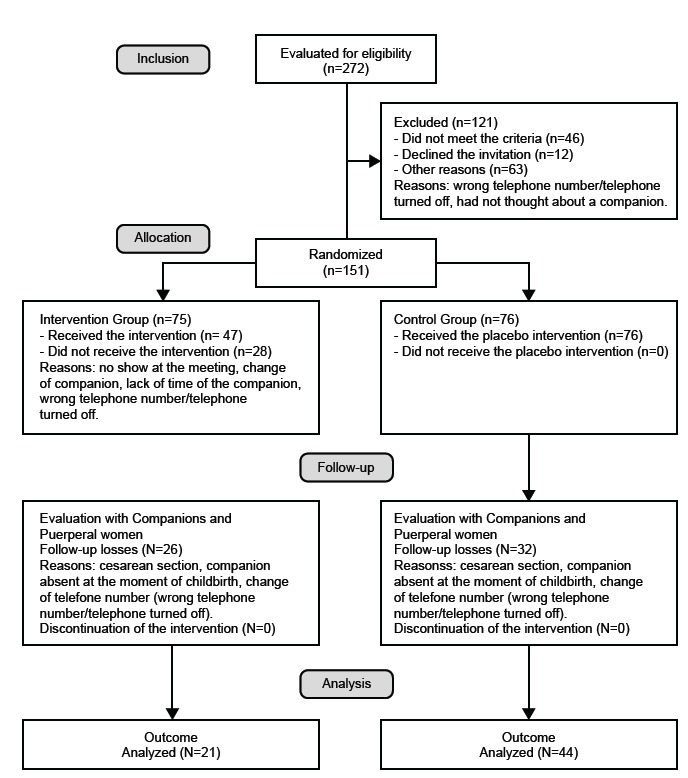
Representative flow chart of participants in each phase of the study as set forth by the CONSORT for non-pharmacological interventions. Fortaleza, CE, Brazil, 2015.

A total of 65 companions and puerperae participated in the study, 21 in IG and 44 in CG. Among the 21 IG companions, 15 (71.4%) were from the CPN-LBC and 6 (28.6%) from the CIESCJLF. Among the 44 companions of the CG, 36 (81.8%) were from the CPN-LBC and 8 (18.2%) from the CIESCJLF. There was no difference between Unit of Origin and allocation group (intervention/control) (Fisher: 0.353). Figure 1 shows the follow-up of participants in each phase of the study. 

Baseline sociodemographic and obstetric data and prior knowledge of the companions on support actions to the parturient and their access to educational activities during the pre-natal care were investigated (Table 1). The companions had, on average, 39.3 (± 14.6) years of age and 8.4 (± 2.5) years of schooling. Among companions, 38 (58.4%) were males, mostly husbands/partners (36; 55.4%), mothers (15; 23.1%) and sisters (8; 12.3%), in this order. There were no differences in these variables between the intervention and control groups.

**Table 1:** Distribution of data of companions according to sociodemographic and obstetrics characteristics and evaluation of prior knowledge about support methods to parturient women. Fortaleza, CE, Brazil, 2015.

Variable	TOTAL (n = 65)		IG (n = 21)		CG (n = 44)		p
	Md (± SD)		Md (± SD)		Md (± SD)		
*Age (years)*	39.3 (±14.6)		41.6 (±12.8)		38.1 (±15.6)		0.562^1^
*Schooling (years)*	8.4 (±2.5)		7.5 (±2.6)		8.8 (±2.4)		0.220^1^
*Income (Brazilian reais)*	1.037.85 (±810.49)		950.22 (±804.47)		1.081.67 (±833.07)		0.699^1^
*Obstetric data*							
Number of pregnancies	2.1 (±1.5)		2.9 (±1.5)		1.8 (±1.4)		0.085^2^
Nº of deliveries	1.7 (±1.3)		2.3 (±1.3)		1.4 (±1.2)		0.076^2^
Nº of abortions	0.2 (±0.5)		0.4 (±0.7)		0.1 (±0.5)		0.375^2^
			N (%)		N (%)		p
*Sex*							
Male	38 (58.5%)		12 (57.1%)		26 (59.1%)		0.882^3^
Female	27 (41.5%)		9 (42.9%)		18 (40.9%)		
*Marital status*							
With partner	49 (75.4%)		15 (71.4%)		34 (77.3%)		0.609^3^
Without partner	16 (24.6%)		6 (28.6%)		10 (22.7%)		
*Degree of kinship with the pregnant woman*							
Husband/Partner	36 (55.4%)		12 (57.1%)		24 (54.5%)		0.213^3^
Mother	15 (23.1%)		7 (33.3%))		8 (18.2%)		
Sister	8 (12.3%)		2 (9.5%)		6 (13.6%)		
Others	6 (9.2%)		0 (4.2%)		6 (13.6%)		
*Questions related to previous knowledge on support actions*	TOTAL (n=65)		GI (n=21)		GC (n=44)		p
	N	%	N	%	N	%	
Participated in preparatory educational activity for childbirth	13	20.0	3	14.3	10	22.7	0.522^4^
Knows support actions to parturient women	44	67.7	16	76.2	28	63.3	0.311^5^
Knows physical support actions	18	27.7	4	19.0	14	31.8	0.282^5^
Knows emotional support actions	22	33.8	16	76.2	27	61.4	0.237^5^
Knows informational support actions	2	3.1	-	-	2	4.5	1.000^4^
Knows negociation/mediation actions	4	6.2	1	4.8	3	6.8	1.000^4^

p1 ^=^ Student’s t-test; p2= ^Wilcoxon^ test; p3 = Chi-square test; p4^=^ Fisher’s exact test; ^p5^= Chi-square test

After participating in the delivery process, the companions were re-evaluated. Table 2 shows the support actions performed by the companions, according to the allocation group.

**Table 2:** Distribution of data of companions according to the types of support provided during labor and delivery. Fortaleza, CE, Brazil, 2015.

Variables	IG (n = 21)		CG (n = 44)		p	RR (95% CI)
	N	%	N	%		
*Support categories*						
Emotional support	56	86.2	20	95.2	0.251^*^	3.21(0.5-21.0)
Physical support	51	78.5	20	95.2	0.026^*^	1.85 (1.03-7.4)
Informational support	6	9.2	2	9.5	1.000^*^	1.85 (0.3-3.4)
Negociation/intermediation	4	6.2	2	9.5	0.589^*^	1.03 (0.8-37.4)
*Support actions*						
Constant presence	19	90.5	34	77.3	0.309^*^	2.15(0.6-8.0)
Words of support	18	85.7	34	77.3	0.522^*^	1.50(0.5-4.3)
Holding the hand	17	81.0	24	54.5	0.039^†^	2.48(0.9-6.5)
Massage	20	95.2	21	47.7	0.001^†^	11.70(1.6-81.8)
Walking	15	71.4	9	20.5	0.000^†^	4.27(1.9-9.5)
On hands and knees position	10	47.6	16	36.4	0.386^*^	1.36(0.6-2.7)
Gym ball	15	71.4	11	25.0	0.000^†^	3.75(1.7-8.4)
Change of position	10	47.6	14	31.8	0.217^†^	1.55(0.7-3.1)
Pray	3	14.3	8	18.2	1.000^*^	0.81(0.3-2.3)
Breathing	15	71.4	7	15.9	0.000^†^	4.88(2.2-10.8)
Shower	3	14.3	9	20.5	0.737^*^	0.73(0.2-2.1)
Guidelines	2	9.5	5	11.4	1.000^*^	0.87(0.3-2.9)

* Fisher’s exact test; ^†^ Chi-square test

Companions who used the educational manual performed a greater number of support actions for parturients (7.2 ± 1.8 in the IG vs 4.6 ± 2.5 in the CG, p: 0.001), being more likely to perform support techniques such as hand holding, massage, walking, gymnastic ball and breathing exercises. 

The experience of accompanying the childbirth was better conceptualized by the participants of the GI, as pointed out by the sum of the items of the Instrument 2. However, the companions of the GI were less satisfied with the way the childbirth took place and with the care provided by health professionals during this moment, as shown in Table 3. 

**Table 3:** Satisfaction of companions according to the evaluation of the experience during labor and delivery. Fortaleza, CE, Brazil, 2015.

Variables	IG (n = 21)		CG (n = 44)		p	RR (95% CI)
	N	%	N	%		
*Birthing labor*						
Satisfaction of being a companion	19	90.5	38	86.4	1.000^*^	1.33 (0.4-4.6)
Satisfaction with the support provided	20	95.2	36	81.8	0.251^*^	3.21 (0.5-21.0)
Satisfaction with the way it took place	16	76.2	42	95.5	0.031^*^	0.38 (0.2-0.7)
Satisfaction with the delay	19	90.5	36	81.8	0.479^*^	1.72 (0.5-6.2)
Satisfaction with the care provided by health professionals	16	76.2	42	95.5	0.031*	0.38 (0.2-0.7)
Usefulness of the support provided	21	100.0	39	88.6	0.166^*^	-
Cooperation with health professionals	19	90.5	36	81.8	0.479^*^	1.72 (0.5-6.2)
*Parturition*						
Satisfaction of being a companion	20	95.2	40	90.9	1.000^*^	1.66 (0.3-9.9)
Satisfaction with the support provided	20	95.2	40	90.9	1.000*	1.66 (0.3-9.9)
Satisfaction with the way it took place	16	76.2	41	93.2	0.100^*^	0.45 (0.2-0.8)
Satisfaction with the delay	19	90.5	37	84.1	0.706^*^	1.52 (0.4-5.4)
Satisfaction with the care provided by health professionals	18	85.7	42	95.5	0.318^*^	0.50 (0.2-1.1)
Usefulness of the support provided	20	95.2	40	90.9	0.148^*^	3.63 (0.5-24.1)
Cooperation with health professionals	19	90.5	36	81.8	0.479^*^	1.72 (0.5-6.2)
*Evaluation Form for Companions in the Delivery Room*	Md (±DP)		Md (±DP)			
Total score	72.43 (±8.18)		64.23 (±7.38)		0.000^†^	-

* Fisher’s exact test; ^†^ Mann-Whitney test

After the evaluation of the companions, the satisfaction of the puerperal women with the process of childbirth was evaluated. The mothers had a mean age of 24.1 (± 6.4) years (24.2 ± 6.2 in the IG vs 23.9 ± 6.6 in the CG, p: 0.796), 8.9 (± 2.3) and 8.9 years of schooling (9.5 ± 2.5 in the IG vs 8.6 ± 2.2 in the CG, p:0.137), and performed on average 7.7 (± 1.6) prenatal consultations (7.4 ± 1.8 in the IG vs 7.8 ± 1.5 in the CG, p: 0.323). There was no difference between the groups for the variables number of gestations (p: 0.278), deliveries (p: 0.060) and abortions (p: 0.428). As to participation in educational activities during prenatal care, 38 (60.3%) responded positively (76.2% in the IG vs 52.4% in the CG, p: 0.069).

Women whose companions were part of the IG had higher means in all the QESC subscales evaluated (Table 4).

**Table 4:** Distribution of means of evaluation of the puerperal women regarding the experience and satisfaction with the process of childbirth. Fortaleza, CE, Brazil, 2015.

Variables	TOTAL	IG	CG	p
	Md (± SD)	Md (± SD)	Md (± SD)	
Subscale 2: Positive Experience	53.4 (±6.2)	55.9 (±6.2)	52.1 (±5.8)	0.034^*^
Subscale 3: Negative Experience^‡^	23.7 (±3.1)	24.8 (±3.4)	23.1 (±2.7)	0.001^†^
Subscale 4: Relaxation	14.9 (±3.4)	17.0 (±3.0)	13.9 (±3.2)	0.002^†^
Subscale 6: Companion’s Support	19.7 (±4.1)	21.8 (±2.3)	18.7 (±4.4)	0.000^†^
Final QESC Score	11.7 (±12.8)	119.6 (±10.4)	107.9 (±12.2)	0.034^*^

* Student t-test; †Mann-Whitney test; ‡Scales with reverse scores, 1.Very much; 2.Fair, 3.Little; 4.None

Women whose companions participated in the GI had greater confirmation of expectations, self-control, self-confidence, knowledge, pleasure and satisfaction with the experience of childbirth (Subscale 2), reported lower levels of fear, malaise and pain (subscale 3), felt more relaxed (Subscale 4) and had a better evaluation of the support provided by the companion (Subscale 5) (Table 4).

## Discussion

The results of this study show that the educational manual is an effective technology to instrumentalize companions to carry out support actions to parturient women, especially actions of physical support. This has a positive influence on the satisfaction of the companions and puerperal women with the experience of accompanying and experience the birth, respectively.

The companions that participated in the study have characteristics similar to those of other studies regarding age, years of schooling, sex, and degree of kinship with the women^11^ ^-^ ^13^. This shows that the sample studied represents well the Brazilian reality. 

The groups did not differ as to the previous knowledge about the support techniques for the parturient women, with emphasis on the reports of supportive actions more present in common sense and those of emotional support. This underscores the importance of health services to offer and encourage the participation of pregnant women and their companions in educational strategies for childbirth preparation, providing counseling, education, trust and support^14^.

 As shown in the flowchart of the study participants, most of the baseline sample was not a companion to the parturient. The main reasons were a change of companion, restrictions of the health service (not acceptance of male companion) and cesarean section without the presence of a companion. Several Brazilian maternity hospitals still do not accept the presence of companions, or accept it partially (during labor only). Among the factors that prevent the inclusion of companions are the non-acceptance by the professionals and the inadequate organizational structure of the services. Specifically in caesarean sections, lack of material resources (dressing and aprons) and increased risk of infection are the main limiting factors^13^.

The findings here show that almost all the companions used some technique to support the parturient, more frequently the techniques of emotional and physical support, in this order. Lack of knowledge is still one of the main barriers to the use of non-pharmacological methods of pain relief among companions^15^. In the comparison of the groups, it was observed that the companions in the GI performed a greater variety of support actions, and were more likely to perform physical support techniques. This indicates the effectiveness of the educational manual for the empowerment of companions and, consequently, for their role in providing support to the parturient. It is worth emphasizing that the educational manual must back the knowledge of the companion regarding the various support actions available, but these actions must be carried out according to the needs of the parturient. 

Participants who used the educational manual evaluated more positively the experience of accompanying childbirth. Among the possible justifications for this finding are: greater satisfaction and feeling of being useful when seing that the support provided increases the well-being of the mothers; less fear and anxiety due to the greater knowledge on the physiology of childbirth, the role of health professionals, and the procedures to be performed (subjects covered in the manual). 

O study brought an unexpected and extremely important finding, going beyond its initial goal. The companions who had access to the educational manual, besides providing more support actions and better evaluation of their experience as companions, also made a more critical analysis of the quality of care provided by the health team. The educational intervention seems to have favored the empowerment of the companions, making them more demanding and questioning, a fact that may justify the greater dissatisfaction of the IG with the health professionals and with the way the labor process occurred. 

Research that investigated the involvement of fathers during pregnancy and childbirth found that those who did not have qualification during prenatal care felt unprepared because they did not know how to help their wives, and impotent because they were mere spectators, they did not understand the work nor their role in this process^16^. In another study, fathers who had access to an educational intervention of preparation for childbirth had a lower risk of experiencing the childbirth event in a frightening way and feeling unprepared for birth^17^. 

The educational manual, besides positively influencing the quality of the support provided by the companions in the delivery room, also contributed to the better evaluation of the women with respect to the experience of childbirth. A similar finding was obtained in a study that investigated the interference of the support provided by companions in the assessment of women regarding the experience of giving birth. The study found that the amount of support provided had a significant association with a positive evaluation of the women^18^. It is worth mentioning that the positive experience of the childbirth process also depends on factors such as availability and accessibility to health services, information and support networks, as well as the model of care provided by health professionals and the adoption of evidence-based practices^19^.

The present study allowed the delimitation of parameters for sample calculation in the definitive study, considering the mean difference in the outcome (number of support actions provided by companions). It was also possible to detect the need for adjustments in the data collection process, in order to minimize the interruption of the losses in the follow-up. It is recommended to ask the participants for a telephone contact of their close relatives in order to help when the attempts to contact the companion/postpartum woman are not successful. It is also necessary to do home visits to the addresses provided in the identification section and/or by Community Health Agents, to interview the participants that could not be contacted by telephone. As a limitation of the study, it is worth mentioning the absence of psychometrically validated Brazilian instruments geared at the evaluation of the support provided by the companion. Another limitation is the disparity in the numbers of participants in the IG and CG.

## Conclusion

The educational manual allowed the companion to provide a greater number and variety of actions to support the parturient. In addition, the use of the manual by the companions had a positive effect on the satisfaction of companions and women with the birthing process. In this sense, the manual is an effective educational technology to be used with this target audience. 

We suggest the realization of further studies to evaluate the effectiveness of other educational interventions that potentiate the abilities of companions as providers of support to the women during the labor process and to evaluate the influence of this support on maternal and neonatal outcomes.
